# Fading connections: A phenomenological study of oncology nurses’ experiences of Missed Nursing Care during an infectious disease outbreak

**DOI:** 10.1371/journal.pone.0336174

**Published:** 2025-11-07

**Authors:** Mahsa Pourshaban, Atefeh Allahbakhshian, Hadi Hasankhani

**Affiliations:** Department of Medical-Surgical Nursing, Faculty of Nursing and Midwifery, Tabriz University of Medical Sciences, Tabriz, Iran; University of Verona, ITALY

## Abstract

**Purpose:**

Providing holistic and high-quality nursing care to oncology patients necessitates integrating care across physical, psychological, spiritual, and emotional domains. This study explored the meaning of oncology nurses’ experiences of missed nursing care during the COVID-19 pandemic.

**Methods:**

This study employed a qualitative design based on Heidegger’s hermeneutic phenomenological approach as the foundational philosophical approach because of its focus on real-life experiences. The sample comprised fourteen nurses employed at various oncology departments in Iran. Data was collected from February 2023 to March 2025 with institutional permission and ethics committee approval (IR.TBZMED.REC.1401.1032). Data were collected through in-depth, semi-structured interviews. Data analysis was conducted concurrently with data collection following the approach outlined by Diekelmann et al. (1989). Standards for Reporting Qualitative Research (SRQR) were used.

**Results:**

Fading connections between oncology nurses and patients, nursing managers, physicians, and the care environment have affected the quality of care and communication during the pandemic. Three subthemes include (1) care behaviors, (2) isolation in duty, and (3) disruption of care canvas and twelve meaning units were obtained. The most care deficits were primarily related to emotional and psychological support.

**Conclusion:**

When human and professional relationships are compromised, the essence of nursing care is lost, and it risks being reduced to a mechanical, task-focused practice. The experiences of oncology nurses during the COVID-19 pandemic reveal significant gaps in nursing care that stem from professional, relational, and systemic challenges. Addressing these issues is crucial for improving nurse well-being and patient outcomes in future healthcare crises.

## 1. Introduction

The COVID-19 pandemic has exerted unprecedented pressure on healthcare systems worldwide, profoundly affecting the delivery of nursing care [1]. Oncology nurses—already subjected to substantial emotional and physical burdens—faced intensified challenges during this period [2]. A pertinent issue that has garnered increasing attention is missed nursing care (MNC), defined as essential nursing actions that are delayed, partially completed, or omitted entirely, which poses a significant threat to patient safety and the overall quality of care [3–5]. MNC poses substantial economic and ethical challenges, necessitating decisions that may conflict with personal and professional values [6].

Numerous studies have examined the prevalence and determinants of MNC across various contexts [7–9] the nuanced lived experiences of oncology nurses—particularly within specific cultural milieus like Iran—remain insufficiently explored [10,11].

Similarly, the Iranian healthcare system, akin to many others, confronted distinctive challenges during the pandemic. Limited resources, escalating patient volumes, and the psychological toll on healthcare workers likely amplified the occurrence of MNC [11,12]. While quantitative approaches can quantify the scope of MNC, they often fall short of capturing the depth and complexity of nurses’ personal and professional experiences [5,13]. In this regard, qualitative research—particularly phenomenological inquiry—provides a vital lens for understanding the essence of these experiences, offering insights that extend beyond numerical data [14].

Extant literature indicates that factors such as nurse burnout, organizational support, and work environment significantly influence the incidence of MNC [15–17]. However, a notable gap exists regarding how these factors interact within the specific context of Iranian oncology nurses during the COVID-19 crisis. Moreover, the moral and ethical dimensions surrounding MNC—especially in environments where nurses confront conflicting demands amid resource scarcity—merit further exploration [18]. The evolving role of oncology nurses, as they adapt to shifting patient needs and incorporate virtual care modalities, adds additional layers of complexity that require deeper understanding [2]. These approaches underscore the multifaceted strategies nurses adopt to deliver holistic care amid challenging circumstances.

This study aims to explore and elucidate the lived experiences of Iranian oncology nurses regarding missed nursing care during the COVID-19 pandemic. Missed nursing care has been widely recognized as a critical factor affecting patient outcomes, yet most studies on this topic have relied on quantitative approaches, which may fail to capture the nuanced, subjective experiences of nurses in high-stress environments. There is a notable lack of qualitative research exploring how oncology nurses perceive and respond to missed care, particularly in the extraordinary context of a global health crisis. Understanding these lived experiences is essential not only for identifying the personal and professional challenges nurses face but also for informing targeted interventions and support mechanisms. By giving voice to oncology nurses’ perspectives, this study aims to contribute to improving care quality for patients and safeguarding the well-being of nurses during and beyond pandemic conditions.

## 2. Aim

This study explored the meaning of missed nursing care in oncology nurses’ experiences during the COVID-19 pandemic.

### 2.1. Research question

What are the characteristics and interpretations of the inherent structure of the lived world or the perception of oncology nurses regarding MNC during the COVID-19 pandemic?

## 3. Methods

### 3.1. Design

This study adopted an interpretive (hermeneutic) phenomenological approach based on Martin Heidegger’s framework to explore the meanings underlying nurses’ lived experiences. This interpretive framework allows for interpreting research elements and exploring the subjective meanings of the individuals involved [19]. We aimed to understand oncology nurses’ experiences regarding MNC during the COVID-19 pandemic rather than extracting their opinions or theoretical viewpoints. Therefore, examining the nurses’ experiences as they were “lived” aligned with the study’s objectives. Standards for Reporting Qualitative Research (SRQR) were used to report the research.

To gain a broader insight, this study was conducted in oncology departments across various hospitals, where there was greater cultural diversity among participants. Oncology departments where adult patients with multiple types of cancer receive chemotherapy or broad-spectrum antibiotics, typically contain one or two isolation rooms. During the COVID-19 pandemic, hematology and oncology patients who tested positive for COVID-19 were occasionally hospitalized in these departments. All patients in the oncology wards were cared for by nurses with at least a Bachelor’s degree. The nurse-to-patient ratio averaged 1:6 during the day and 1:10 during night shifts. Each room consisted of 2–6 beds.

The research involved qualified interviewers with doctoral degrees in nursing and over ten years of experience. They received specialized training to enhance their interview techniques and understanding of ethical principles. Many had prior qualitative research experience, creating a supportive environment for participants. To ensure interview effectiveness, two pilot interviews were conducted. Feedback from these sessions led to discussions among the research team to address potential issues. These qualifications and the iterative refinement process significantly enhance the credibility and quality of the data collected. The interviewer was mindful of possible biases and assumptions related to participants’ experiences. They also had a strong interest in the research topic.

### 3.2. Participant

Oncology nurses from inpatient departments (N = 14) were recruited using purposive sampling, aiming to include participants with diverse demographic and professional characteristics to achieve maximum variation. Eligible nurses had at least six months of experience providing care to oncology patients during the COVID-19 pandemic, could communicate effectively, and were willing to share their experiences related to the research topic. To ensure depth and richness of the data, arrangements were made to conduct 28 interviews with 14 participants. Sampling continued until no new codes emerged, and data saturation was reached. Purposive sampling methods primarily emphasize saturation, a core concept in interpretive research, meaning that data collection continues until no new relevant information emerges from additional interviews [20]. Participants did not refuse or withdraw at any point. Table 1 presents the demographic details of participants, including age, gender, marital status, number of children, education level, work experience, unit, shift type, and interview durations.

**Table 1:** Interview guide and sample probing questions.

Main questions	Probing questions
What areas of care did you spend the most energy in during the COVID-19 pandemic?	Could you describe a specific situation that reflects this? Why do you think these areas required more effort?
Can you share an experience or example from the COVID-19 pandemic when nursing care was missed?	What factors contributed to this missed care? How did you feel in that situation? What was the outcome for the patient?
Please explain the types of care that were less feasible to perform during the COVID-19 pandemic.	What made these types of care difficult to deliver? How did you manage or compensate for these limitations?
–	Additional probing questions were used throughout the interviews to gain deeper insights, such as: “Can you give an example?”, “Please explain more about that”, “How did that make you feel?”, or “What do you mean by that?”

### 3.3. Data collection

The data were collected through in-depth interviews. The interviews were conducted face-to-face in two sessions with each participant, for an average of 36 minutes per session. Data collection was conducted from February 2023 to March 2025. A total of 32 interviews were conducted, including 4 pilot interviews with 2 participants that were excluded, leaving 28 interviews involving 14 participants for the main analysis. To achieve data saturation, an extra 4 interviews were conducted. Since signs of repetition appeared around the 24th interview, these additional sessions helped confirm saturation and strengthen the study’s findings. The interviews were conducted with informed consent and without imposing bias on the participants. Participants’ privacy was protected by using pseudonyms, storing data securely, and ensuring that no identifying information appeared in the transcripts or reports. Before starting the interviews, participants were contacted by phone to ask preliminary questions, which helped them select suitable candidates and determine the interview time and location. Interviews were conducted in a private room within the oncology wards, ensuring a quiet and comfortable environment. Before each interview, the researcher explained the purpose and significance of the study to the participants and assured them of confidentiality to build trust and encourage open communication. At the beginning of each interview, informal questions were used to “unfreeze’. Demographic information was gathered, including gender, age, marital status, education, work experience, and shift type during the pandemic. The interview questions were developed based on the study objectives and the philosophical underpinnings of Heideggerian interpretive phenomenology. A review of the relevant nursing literature on missed nursing care and nurses’ experiences during the COVID-19 pandemic informed the content of the questions. Open-ended and exploratory questions were designed to elicit rich, in-depth narratives about participants’ lived experiences. Three qualitative research experts reviewed the interview guide to ensure clarity and relevance. Table 1 presents examples of the main and probing questions used in the semi-structured interviews.

All the interviews were audio-recorded and transcribed verbatim. Data saturation was established using a systematic approach, where interviews were conducted until no new themes or significant insights emerged. This point was reached after the 24th interview, as responses became repetitive and aligned with earlier data. Ultimately, 28 interviews involving 14 participants were included in the analysis. The collection was concluded once recurring themes were consistently identified and no new information appeared, indicating that a thorough understanding of oncology nurses’ experiences had been achieved.

### 3.4. Data analysis

Data analysis was conducted concurrently with data collection, following the seven-step process outlined by Diekelmann et al. (1989), grounded in Heideggerian phenomenology. This process began with meticulous transcription of interviews, followed by multiple rounds of review to develop a comprehensive understanding of the context. Each transcript was interpreted to uncover underlying meanings through systematic coding. Team discussions facilitated the identification and evolution of themes, leading to the emergence of new issues. This iterative dialogue among team members allowed for the extraction of both explicit and implicit meanings from the interview texts, encompassing participants’ verbal statements and the overall atmosphere of the interviews.

The primary objective of data analysis was to derive concepts and identify constitutive patterns that encapsulated the central meanings, similarities, and differences of the concepts. Throughout the analysis, a combined analytical approach was employed, synthesizing interpretive summaries to connect identified themes effectively. A draft version of the themes, accompanied by selected excerpts from the interviews, was presented to the analysis team and individuals familiar with the research methodology. Reflexive analysis involved deep contemplation and iterative reflection, emphasizing a hermeneutic movement from parts to the whole and vice versa at all stages of the analysis.

### 3.5. Trustworthiness

In this study, adherence to the reliability criteria of credibility, dependability, confirmability, and transferability was ensured in accordance with Lincoln and Guba’s (1985) framework [21].

*Credibility:* The interviews were transcribed promptly by the researcher, with concurrent external review undertaken by the research team at each stage of analysis. Continuous engagement with participants contributed to the validation of identified themes. All interviews were documented in both written and audio formats, with careful measures taken to ensure confidentiality and ethical handling of the data. Participants were assured that their information would be evaluated objectively and impartially, and strict confidentiality was maintained throughout the research process. Furthermore, the alignment of derived themes with current literature in nursing research relevant to the topic was regularly assessed through ongoing data analysis and participant interaction*. Dependability:* A pilot study involving two participants was conducted to evaluate the clarity and relevance of the semi-structured interview questions, ensuring their suitability for addressing the research objectives. *Confirmability:* Feedback from the research team regarding the appropriateness of the interview questions and their relevance to the phenomenon under investigation was solicited to support objectivity. Transferability: Detailed demographic data of the participants were collected and systematically described. Additionally, the findings were shared with non-participating nurses to facilitate comparison and enhance contextual transferability. Data coding and theme extraction were managed using MAXQDA software (2020 version).

### 3.6. Ethical considerations

This article was derived from a specialized doctoral thesis in nursing, which has received approval under the code IR.TBZMED.REC.1401.1032 (Approval Date: 2023.06.02). Permission to access the research environment was also obtained. At the study’s outset, informed and written consents were obtained from each participant. Participants were provided with necessary explanations regarding the procedures and objectives of the study as well as their right to withdraw from participation at any time and in any aspect of the research. Consent was also sought to record the interviews. To uphold the principles of confidentiality and privacy, the names of the participants remained anonymous throughout all stages of the research, and measures were taken to avoid disclosing any information that could lead to the identification of the interviewees. Accordingly, each participant was assigned a unique code. The Declaration of Helsinki complied with all steps of the study.

## 4. Results

### 4.1. Participant’ characteristics

Table 2 presents the participants’ key demographic characteristics. The average age of the group was 38 years, comprising 9 female and 5 male participants. All participants held a university degree, with most having a Bachelor’s degree. The average duration of work experience as a nurse is approximately 11 years.

**Table 2:** Demographic characteristics of the participants.

Code	Age (years)	Gender	Marital status	Education	Work experience (years)	Unit	Shift type
P1	30	Female	Single	PhD student	6	ICU (oncology-related)	Night
P2	46	Male	Married	Bachelors	18	Oncology ward	Rotating
P3	29	Female	Single	Bachelors	6	Oncology ward	Night
P4	32	Male	Single	Bachelors	8	Oncology ward	Evening- Night
P5	52	Female	Divorce	Bachelors	20	Oncology ward	Morning
P6	36	Female	Single	Bachelors	3	Oncology ward	Morning-Evening
P7	40	Male	Married	Bachelors	9	ICU (oncology-related)	Rotating
P8	43	Male	Married	Masters	15	Oncology ward	Morning
P9	34	Female	Married	Bachelors	7	Oncology ward	Rotating
P10	47	Female	Married	Bachelors	16	ICU (oncology-related)	Morning- Evening
P11	28	Male	Single	Masters	4	ICU (oncology-related)	Night
P12	39	Female	Married	Bachelors	14	Oncology ward	Night
P13	36	Female	Married	Masters	10	Oncology ward	Evening
P14	41	Female	Married	Masters	17	Oncology ward	Night

### 4.2. Main theme

**Fig 1:**
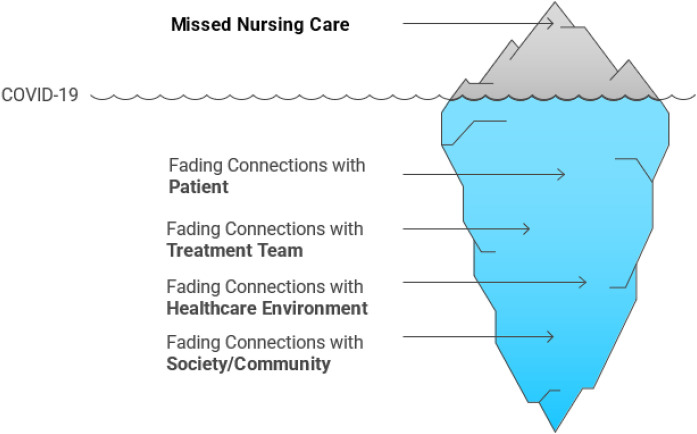
Fading Connections and Missed Nursing Care during the COVID-19 pandemic in oncology departments.

The theme of “fading connections” captures the weakening or disruption of human and professional relationships in nursing during times of crisis, ultimately compromising the delivery of care. In the nursing context, interactions with patients, colleagues, healthcare teams, the community, and the care environment are not merely routine tasks—they form the foundation of professional experience and nursing identity. During the COVID-19 pandemic, however, these essential connections were profoundly challenged and resulted missed nursing care in oncology departments during the COVID-19 pandemic (Fig 1).

This theme reflects the lived experiences of nurses who, constrained by structural limitations, infection control measures, social distancing, resource shortages, high workloads, and anxiety about personal risk, were compelled to limit their human and professional interactions. While these constraints may superficially appear as a mere “reduction in communication,” they fundamentally signify a disruption in the nurse’s presence in the world of care, as human connection is inseparable from “being a nurse.” The overarching theme of “fading connections” comprises three subthemesand twelve meaning clusters, each illustrating a distinct dimension of this relational breakdown. Information regarding the main theme, sub-themes, and meaning clusters is presented in Table 3.

**Table 3:** Main theme, sub-themes and meaning clusters obtained from interpretative phenomenological analysis of interviews by oncology nurses in Iran.

Main Theme	Subthemes	Meaning Cluster	Quotes
Fading Connections	Missed Care Behaviors	Compulsory omission	“Because of COVID, my workload got really heavy, and there was never enough time. Most of the time, I had to skip some care, like talking with patients, just to make sure everything got done according to schedule. I felt like I didn’t have enough time to do what I really wanted for my patients.”
		Shortened care	“Because of the risk of COVID, I had to rush through care, since I was afraid of the virus—both for myself and for my patients who were in special conditions. On top of that, I hardly had time to connect with my patients beyond the essential tasks, and all of this made me miss the moments when my patients really needed me.”
		Task-oriented care	“During COVID, I felt that if I tried to complete all the care tasks, I wouldn’t be able to care for all my patients. Saving lives and keeping my patients stable were my top priorities. I had to focus on the essential and critical tasks first.”
		Interrupted care	“During COVID, sometimes care would be incomplete because of emergencies with some patients. For example, I might be giving medication when suddenly another patient’s condition worsened, and I had to leave quickly, leaving my task unfinished.”
		Delayed care	“During COVID, I tried to get all my tasks together so I could go into the patient’s room just once, finish everything there, and then leaves. Because of this, medication timing or care schedules might get changed or shifted.”
	Isolation in Duty	Insufficient backing	“During that period, I felt lonely. There was no support from supervisors. Once, a patient needed suctioning, but because we weren’t given enough protective equipment, I couldn’t go to the bedside as often as needed. I felt abandoned. Even the head nurse wasn’t supportive. It was like everyone expected me to just cope with the situation.”
		Imbalance in power	“During COVID, as a nurse, I knew my patient’s needed things like isolation, but the doctors didn’t accept my input and would admit COVID patients. This made me feel like my care and concerns didn’t matter. I felt I had no control over the patients or the ward, like I had no power to make real changes.”
		Stigmatized isolation	“During COVID, society saw us nurses as disease carriers, which isolated us emotionally and socially. While providing care, I was constantly reminded that I shouldn’t forget my own safety first. This made delivering compassionate care even more difficult.”
		Nurse unmet needs	“Because of the long hours and dealing with COVID without extra pay, we were financially strained, and our safety wasn’t really considered. For example, there was no proper protective equipment or protocols—it was like no one truly understood what we needed, and there was no emotional support from management. On top of that, many procedures required training, but there wasn’t enough guidance or support.”
	Disruption of Care Canvas	Storm of responsibilities	“At the peak of COVID, I felt trapped in my responsibilities and had to try to balance caring for patients, my own fears, and the chaos in my duties. It was like every new day brought a new wave of responsibilities that I had to get through.”
		Intensified environmental issues	“When COVID came, it felt like the ward was suffocating. The space was inadequate, protocols were strict, and staffing was insufficient. When everything was so limited and tense, even worse than before and changed, connecting with patients and completing all care became more difficult. The environment was full of anxiety.”
		Forced adaptation	“During COVID, I had to use protective gear to keep myself safe from the virus. Putting on the protective gown took time, and when it slowed me down, I couldn’t get to all the care tasks on time.”

#### 4.2.1. Care behaviors.

The sub-theme care behaviors reflect nurses’ lived struggle between their professional ideals and the constraints imposed by crisis conditions. The five meaning clusters—*compulsory omission, shortened care, task-oriented care, interrupted care, and delayed care*—reveal that missed care was not merely a set of neglected actions but an existential phenomenon rooted in the conflict between being able and being forced. Nurses’ descriptions uncover a moral and emotional dimension where omission, delay, and interruption were experienced as wounds to their professional integrity and identity. From a hermeneutic perspective, these accounts illuminate the essence of “being a nurse in crisis” as a continual negotiation between ethical responsibility and situational impossibility. The meaning of missed care, therefore, transcends the operational level—it becomes a manifestation of the nurse’s being-in-tension: caught between the duty to care and the impossibility of fully realizing that duty amid a collapsing care world. Time and resource constraints, along with the obligation to adhere to infection prevention protocols or the fear of contracting the virus, create a conflict between professional responsibilities and the nurse’s actual capabilities. Consequently, nursing care is compromised in various ways.

**4.2.1.1. *Compulsory omission:*** During the pandemic, nurses frequently encountered moral and professional dilemmas that forced them to omit parts of care. These omissions were not rooted in negligence but emerged from overwhelming workload, resource scarcity, and competing priorities. Nurses described the emotional burden of these unavoidable choices and the resulting guilt and moral distress. The following quotations illustrate how workload intensity and staffing shortages forced nurses to prioritize certain care tasks over others:

*“During the pandemic, I had to skip some tasks or care conversations with patients just to finish work on time. There was never enough time to do everything we wanted.” (P4)*

*“Heavy workload meant I sometimes neglected routine care or skipped talking to patients, because there wasn’t enough time.” (P14)*

These reflections reveal that omission became an existential experience of moral dissonance, where nurses struggled between their professional ideals and the limitations imposed by the crisis. Missed care thus transformed from a managerial issue into a lived moral conflict of being unable to care as one wishes.

**4.2.1.2. *Shortened care:*** This meaning cluster captures how infection control measures, particularly for vulnerable oncology patients with weakened immune systems, have led to a reduction in the duration and scope of care provided by nurses and reshaped the nurse–patient relationship. Fear of contagion, limited time, and safety protocols shortened the depth and emotional content of care encounters, turning intimate moments into quick, functional exchanges. The following quotations illustrate how infection prevention protocols restricted nurses’ direct engagement with patients:

*“My communication with patients became very limited. I tried to spend less time near them and do only what was necessary to reduce exposure.” (P6)*

*“To avoid any risk of infection, I kept my time at the patient’s bedside short and avoided unnecessary contact, even if I knew it meant providing less support for their needs.”(P11)*

*“Even if I had time, I preferred not to talk over the patient’s bedside. Even brief conversations while giving medication made me anxious—both about getting infected myself and about the patient catching it.” (P7)*

This indicates that stringent safety precautions, particularly for immunocompromised patients, have significantly reduced the duration and intimacy of care, resulting in a decline in the overall quality of nursing support. In this context, shortened care was not simply reduced time but represented a loss of presence — an existential distancing where nurses’ caring selves were constrained by fear and precaution.

**4.2.1.3. *Task-oriented care:*** Under immense pressure, nurses shifted toward prioritizing technical and survival-focused duties over holistic or psychosocial aspects of care. The focus moved from “being with” to “doing for,” reflecting a pragmatic response to crisis conditions. The following accounts illustrate how nurses concentrated on immediate patient needs while deprioritizing less urgent tasks:

*“Because oncological patients were very dependent on their medications. The patient’s hemodynamic status, vital signs, and medication management were really important to me. After that, if I had time, I would address issues like dressing changes, change position, and gavage feeding.”* (P1)

*“During the pandemic, we faced tough realities and lost hope for our patients. I had to adjust my priorities in that critical situation to offer the best possible care. So, I focused on my patients’ more urgent and vital needs.”* (P9)

*“My interaction with patients was very limited. I carried out care that required close bedside presence very selectively. I focused first on addressing life-threatening issues.”* (P8)

This pattern reveals a transition from relational nursing to instrumental performance, reflecting an ontological shift from “caring presence” to “task execution,” where nurses negotiated their moral identity within systemic limitations.

**4.2.1.4. *Interrupted care:*** This meaning cluster explores how systemic pressures, emergencies, and rapidly changing priorities during the pandemic often disrupted the continuity of oncology care. Interruptions in care reflected the chaotic, unpredictable reality of the oncology ward during the pandemic. Emergencies, sudden patient deterioration, and staff shortages disrupted the continuity of care, leaving nurses distressed about incomplete treatments. The following excerpts illustrate these disruptions:

*“Many treatments were interrupted or postponed unexpectedly, which we knew could negatively affect the patients’ recovery.” (P12)*

*“For example, I might be giving a chemotherapy injection to a patient, and then because of a cardiac arrest or a severe drop in oxygen in another patient, I would have to leave everything. When I came back, I’d see the patient’s IV disconnected or the medication left unfinished, and it was very upsetting because I knew these interruptions affected the patient’s recovery.”(P5)*

*“While giving chemotherapy, I often had to stop in the middle of treatment because of an emergency with another patient. As a result, oncology care was often left unfinished, and the quality of chemotherapy was compromised.” (P11)*

These accounts reveal how interruptions were a constant feature of care delivery during the pandemic, reflecting the unpredictability of clinical demands and the emotional strain of leaving critical tasks incomplete. The dynamic and unpredictable environment during the pandemic, where nurses had to shift rapidly from one patient to another, resulted in incomplete treatments and potential risks for patients due to missed interventions. Such interruptions were not mere workflow issues but embodied the experience of fragmented caring — a breakdown in the temporal flow of nursing that intensified feelings of powerlessness and ethical discomfort.

**4.2.1.5. *Delayed care:*** Delays occurred when infection control priorities and emergency duties forced nurses to postpone non-urgent procedures. These deferrals, though pragmatic, often created anxiety about compromising patients’ safety. The following statements illustrate how nurses managed these competing priorities:

*“I postponed monitoring vital signs and blood sugar levels, delaying procedures like suctioning a COVID patient with tracheostomy to reduce transmission risk.”* (P10)

*“Some routine tasks had to be put off until later because urgent needs took priority.”* (P3)

*“My patient was undergoing chemotherapy, receiving anti-nausea medication, and also needed a dressing change due to surgery. Because of risk of COVID, I had to do all these tasks back-to-back in one visit. This often-caused delays in the anti-nausea medication, chemotherapy, or dressing change, which negatively affected the patient’s overall condition.”* (P5)

Infection prevention measures, aimed at reducing exposure risks, often result in delays in essential routine assessments and potentially postpone the early detection of complications. Delayed care symbolizes the temporal struggle of nurses caught between urgency and compassion — an existential negotiation between doing what is possible and what is ethically desired.

#### 4.2.2. Isolation in duty.

This sub-theme includes four meaning clusters: *insufficient backing, imbalance in power, nurse unmet needs, and stigmatized isolation*. The sub-theme Isolation in Duty encapsulates the ontological experience of separation — from colleagues, from leadership, from society, and even from one’s own caring self. This isolation was not only physical but deeply existential, symbolizing a loss of mutual understanding that underpins authentic nursing care. The nurses’ voices show that missed care emerged not only from external constraints but also from the inner erosion of relational connectedness — a fading of the very being of the nurse as “one who cares”— a profound feeling of being left alone with overwhelming responsibility. Participants described an environment where insufficient managerial support, hierarchical power relations, unmet needs, and social stigma collectively eroded their sense of belonging and professional worth. This isolation affected their emotional well-being and the integrity of their care, creating a deep disconnect between their professional ideals and lived reality.

**4.2.2.1. *Insufficient backing:*** During the pandemic, nurses often felt abandoned by supervisors and administrators who were expected to provide guidance, empathy, and tangible assistance. Instead, participants described a void of leadership presence that left them feeling unprotected and unseen. The lack of encouragement and understanding undermined their morale and further intensified the emotional toll of their work. The lack of adequate support diminished their ability to provide comprehensive care, resulting in certain nursing tasks and activities being overlooked or performed insufficiently. The following quotations illustrate how insufficient supervision and lack of teamwork diminished nurses’ capacity to deliver optimal care:

*“Our supervisors avoided checking on the departments and visited less often. I thought, they’re scared, but I’m facing this full-time. With the same salary, they may earn more and have lighter workloads. This affected my morale, leading to less effort and lower care quality.”* (P5)

*“My patient became unwell during plasma exchange, and I couldn’t complete the treatment for my other patients. When the head nurse arrived in the morning, instead of support or appreciation, she asked why I hadn’t finished the plasma exchange. I explained that I hadn’t eaten and had been on my feet all night; I was exhausted. This lack of understanding affected my motivation at work.” (P4)*

*“Sometimes, my colleagues acted selfishly, saying they wouldn’t follow up if I didn’t manage. If they had shown more compassion and checked the previous shift’s work, it could have helped the patient and reduced harm. My colleagues were concerned and preferred not to be in close contact with a COVID patient.”* (P1)

The narratives portray insufficient backing as more than a management issue; it reflects a moral breakdown in the caring culture of the institution. Nurses experienced the absence of leadership not merely as operational neglect but as an existential abandonment that diminished their sense of professional being and purpose.

**4.2.2.2. *Imbalance in power:*** Nurses’ accounts revealed that hierarchical structures within hospitals became more rigid during the pandemic. Many participants felt their professional voices were dismissed, particularly in decision-making about patient care and infection control. When nurses feel that their voices are unheard or their power is constrained, they are less likely to advocate for their patients, which can negatively affect the quality of care. Such inequalities hinder effective communication and collaboration, thereby compromising decision-making and resulting in incomplete or suboptimal patient care. The following quotations illustrate the effects of power imbalances on nurses’ practice:

*“Surgeons, despite knowing the patient was positive, prioritized their interests and forced us to care for COVID patients in the oncology unit, which was wrong. With limited authority as a nurse, I stopped putting in extra effort because my opinions didn’t matter, leaving me powerless for myself and the innocent patient.”* (P10)

*“I wanted to always work with my patients without rushing, to talk with them, and spend more time with them. I wanted care to be complete. But I felt that if I spent too much time, I would get negative feedback from the head nurse or shift supervisor, and my authority was limited. Because of this, I sometimes had to skip some care details. With limited time and a high patient load, focusing on quantity over quality seemed more important to the management.” (P6)*

A perceived lack of autonomy and concern about negative feedback prevent nurses from fully exercising their professional roles, even when they possess strong intentions and motivation to provide comprehensive care. These constraints not only diminished the quality of patient care but also reinforced nurses’ feelings of inefficacy, stress, and reduced professional motivation. These experiences illuminate the imbalance in power as a deeply lived experience of disempowerment and moral silence. Nurses’ inability to influence care decisions undermined their sense of authenticity and created a dissonance between their ethical commitment and institutional reality — an ontological experience of “being powerless to care.”

**4.2.2.3. *Nurse unmet needs:*** Participants emphasized that during the pandemic, their fundamental professional and human needs — safety, education, emotional support, and fair compensation — were neglected. These unmet needs profoundly affected their ability to sustain quality care, comprehensive, safe, and effective care and emotional resilience. The following quotations exemplify the multifaceted nature of unmet needs:

*“The most challenging part of nursing was the nursing office. The supervisors had an inappropriate attitude, often giving vague responses and showing little concern for our requests for personal protective equipment. The head nurses and other staff members’ lack of support and empathy affected my care. Even a calming word from them would have helped me to perform better. If the supervisors were willing to help, it would have positively impacted my work quality.”* (P4) This highlights how unmet safety needs and the absence of emotional support from management diminished nurses’ capacity to deliver safe and effective care.

*“During COVID, we often needed psychological support, as we would cry for weeks over patients who had passed away. The emotional burden on oncology nurses was significant, and the added stress of COVID greatly impacted our ability to provide care.”* (P8) This underscores that unmet emotional and psychological needs increased emotional exhaustion, which compromised nurses’ focus and their ability to maintain high standards of patient care.

*“I needed to improve my knowledge about COVID-19 and oncology, especially since laryngospasm—a side effect of Eloxatin—can mimic COVID-19 symptoms. There should have been training for nurses, but there wasn’t.”* (P6) This indicates that unmet educational needs hindered nurses’ competence, increasing the risk of misdiagnosis and suboptimal patient management, thereby affecting care quality.

*“Payments were often delayed, and since I worked in the oncology department, we didn’t receive COVID allowances. The compensation was minimal; even the nursing supervisor believed we weren’t a COVID unit. However, many of our cancer patients were COVID-positive. This situation impacted my morale and motivation, causing me to provide less care, as I felt this burden was unfairly imposed on us. The biggest motivation is financial, and that can’t be denied.”* (P2) This reflects how unmet financial needs and feelings of undervaluation led to decreased motivation, further reducing the quality and consistency of nursing care. Overall, these unmet needs—whether related to safety, emotional well-being, education, or finances—had a cumulative impact on diminishing nurses’ ability and motivation to deliver optimal care, ultimately compromising patient safety and care standards during the pandemic. Here, unmet needs are not merely organizational deficiencies but existential wounds to the nurses’ professional identity. The absence of recognition, support, and security disrupted their being-in-care, leading to feelings of invisibility, fatigue, and moral exhaustion — states that lie at the heart of the phenomenology of missed nursing care.

**4.2.2.4. *Stigmatized isolation:*** Beyond the workplace, nurses experienced social exclusion and stigma from the community and even from family members. Their identities as caregivers became paradoxically associated with contagion and danger, leading to emotional alienation and psychological distress. Social exclusion and internalized fears have led to emotional distress, reduced social support, and heightened feelings of loneliness, which in turn have hindered nurses’ focus and motivation, ultimately affecting the quality of patient care. The following quotations highlight nurses’ experiences of stigmatized isolation:

*“When I went to the pharmacy and mentioned we had a COVID-19 patient, the staff member backed away. I reassured them, but this distance from people affected me. Even ride-sharing services would not come to the hospital yard because of contamination fears, which made me feel bad. As a result, I tried to limit my interactions with suspicious patients and protect myself more.”* (P7)

*“My connection with friends and family had become limited. Not only did I think I was a virus carrier, but I could also sense fear in their expressions. It seemed like they thought I was a virus carrier too. These thoughts stayed with me throughout the shift, and I also thought about taking care of myself.”* (P13)

These reflections demonstrate how social stigma extended nurses’ professional isolation into their personal lives. The stigmatized isolation reveals a rupture in their sense of belonging and recognition — transforming their caring identity into one marked by fear and exclusion. In phenomenological terms, nurses’ being-with-others was profoundly altered, turning everyday relationships into reminders of vulnerability and estrangement and potentially impaired their ability to provide continuous and compassionate care to patients.

#### 4.2.3. Disruption of care canvas.

The care environment is not merely a physical space but an existential setting in which nursing practice acquires meaning. During the pandemic, drastic changes in the physical and organizational structure of the workplace—including patient overcrowding, equipment shortages, constant unit reassignments, and time pressures—led nurses to feel that their environment had become uncontrollable and unfamiliar. This experience fostered a sense of alienation within the world of care. The sub-theme Disruption of Care Canvas embodies the collapse of the familiar world of nursing. The “canvas” — the relational, spatial, and moral background on which care unfolds — was torn by the pandemic’s pressures. The storm of responsibilities fragmented time, environmental constraints distorted space, and forced adaptation disrupted embodied communication. Together, these ruptures signified a breakdown of the lifeworld of care — a loss of coherence in the nurses’ experience of “being-in-care.” From a hermeneutic viewpoint, this disruption represents more than situational stress; it reflects a transformation in the nurses’ ontological relationship with their work. Their caring presence was reframed by survival imperatives, replacing the rhythm of compassion with the mechanics of endurance. In this fractured world, the essence of care persisted only as a fading echo of its former wholeness. This sub-theme encompasses three interrelated meaning clusters: *storm of responsibilities, intensified environmental issues, and forced adaptation*. Participants indicated that the overwhelming burden of increased responsibilities, coupled with an environment that failed to sufficiently adapt to the new virus conditions and faced shortages of human and material resources, severely disrupted the delivery of nursing care. As a result, nurses were compelled to adopt new practices and coping strategies, which often led to compromises in care quality. Heightened workplace constraints affect nurses’ professional experiences during crises, leading to the missed nursing care.

**4.2.3.1. *Storm of responsibilities:*** Nurses described how the pandemic’s surge in duties created a relentless storm of demands, leaving them struggling to maintain control over competing tasks. Simultaneous crises, staff shortages, and the influx of new patients overwhelmed their capacity to deliver consistent care, resulting in missed nursing interventions and potential deterioration in patient outcomes. The following quotations illustrate the impact of overwhelming responsibilities on care delivery:

*“There were times when, for example, a patient would become critically ill, have various tests, experience bleeding, or face an acute problem. The workload was so high that I didn’t have time to follow up on these issues at all. Then we would see that by the next shift or the next day, the patient condition had worsened significantly, and everything had fallen apart; she/he had been intubated and had faced several other problems.”* (P1)

*“Because of staff shortages, nurses from other departments were sent to our unit, but they hadn’t received any training in chemotherapy or oncology care. It was our responsibility to teach them, which took up a lot of our time, leaving us with no time for other types of care.”* (P14)

These experiences indicate that heavy workloads and urgent, unpredictable responsibilities made continuous monitoring and responsiveness to patient needs challenging for nurses, leading to lapses in nursing care and deterioration of patient outcomes. In this storm of responsibilities, the nurses’ experience reveals an existential sense of being overwhelmed. Their professional rhythm collapsed under the weight of simultaneous urgencies, producing an inner fragmentation that mirrored the external chaos of the ward. The temporal flow of care—so central to the nurse–patient relationship—was lost in an ongoing crisis of prioritization.

**4.2.3.2. *Intensified environmental issues:*** Participants emphasized how deficiencies in infrastructure, space, and supplies intensified the challenges of care and hindered proper infection control and patient management. Working in overcrowded oncology wards, lacking protective equipment, and managing both COVID-positive and negative patients created constant tension and often forced oncology nurses to prioritize certain tasks over others. The environment itself became an obstacle to caring. The following quotations illustrate the challenges posed by environmental constraints:

*“The infrastructure to keep non-COVID patients separate from COVID patients on the same unit wasn’t enough. We also didn’t have enough staff. Because of that, infection control could get tricky—one nurse might have both a COVID-positive and a COVID-negative patient on the same shift. All of this meant that, as a nurse, I sometimes had to forgo certain care tasks for some of my patients in order to control infections.”* (P3)

*“We faced limited time, resources, and inadequate space. There was a shortage of professional masks, and while some latex gloves were available, they weren’t abundant. The patients’ antibiotic volumes had gone up because of COVID, and it took a lot of time. We also didn’t have enough isolation rooms.”* (P5)

“Everything changed overnight. It felt like I had entered a completely different world where nothing was familiar anymore. You just had to survive and keep going.” (P7)

This meaning cluster exposes how the care environment—usually a space of safety and healing—transformed into a site of anxiety and risk. From a phenomenological lens, the spatial world of nursing shifted from dwelling to enduring. Nurses’ lived space was no longer supportive but constraining, symbolizing the breakdown of harmony between the caregiver and the environment. In this situation, the nurse experiences a rupture in them. The work environment that was once familiar now becomes alien and uncertain. This sense of estrangement gives rise to feelings of insecurity, existential anxiety, and a distancing from the authentic act of caring.

**4.2.3.3. *Forced adaptation:*** The pandemic required nurses to continually modify their practices, often at the expense of relational and emotional aspects of care and impacted the effectiveness of care. Wearing layers of PPE, maintaining physical distance, and navigating new safety routines redefined how they related to their patients. These adaptations, while necessary, created feelings of alienation from their usual caring self. The following quotations illustrate how forced adaptations altered nurses’ practice:

*“I had to gauge the patient’s emotional state through their eyes due to the mask. Early on, I would check in with patients from the door or behind glass. This made it difficult to assess my patients properly. I was less able to identify my patients’ emotional needs, and as a result, the emotional support they received was lower.”* (P8)

*“Washing hands and changing personal protective equipment between patients was tedious and time-consuming. However, it was crucial for cancer patients, as they often had low immunity. Unfortunately, this meant that a lot of time was lost, and many other aspects of care were left unfinished or not done at all.”* (P12)

In this forced adaptation, nurses’ being-with-patients was fundamentally altered. The body, which is the medium of care and empathy, became shielded and restricted. What emerged was a paradoxical experience: to protect life, nurses had to distance themselves from it, leading to a deep sense of disconnection and loss of authenticity in their caring practice. The strict PPE protocols and protective measures in the care of cancer patients was time-intensive, resulting delays, interruptions and omitted essential aspects of patient care, underscoring the challenge of balancing safety with comprehensive nursing support.

## 5. Discussion

This study elucidates the connections and experiences of nurses who worked in oncology and experienced MNC during the COVID-19 pandemic. According to the participating nurses, poor communication was prevalent across various dimensions during this period, and reflect systemic and emotional challenges impacting care quality. It was clearly noted that deficiencies in the care environment were a significant cause of MNC. The requirement for social distancing to control infections, were also contributing factors to the MNC. These findings suggest that MNC is a multifaceted issue requiring holistic interventions, aligning with prior research calling for systemic changes to support oncology nurses during pandemics [22].

### 5.1. Missed care behaviors

The sub-theme of missed care behaviors—namely, enforced omission, care shortening, task-oriented care, delayed care, and discontinued care—closely align with existing definitions of missed care throughout the pandemic context. Safdari and colleagues identified hospital nurses’ care during the pandemic as including care provided at inappropriate times, incomplete care, interruptions in care, and improper care [12]. Oncology nurses frequently omitted care due to overwhelming workloads and time constraints, a phenomenon well-documented in nursing literature. Falk et al. reported that staffing shortages and high patient acuity lead to care omissions [23]. Similarly, Albsoul et al. noted that time pressure forces nurses to prioritize essential tasks, often neglecting psychosocial support [24], aligning with participants’ reports of skipping patient conversations. The necessity to restrict interactions due to infection control measures is further supported by Sugg et al., who observed that PPE protocols and nurse’ reluctance to spend extended time with patients out of fear of COVID-19 transmission led to decreased direct patient contact, consequently undermining the comprehensiveness of care [25]. During COVID-19 pandemic, fears of infection and the necessity of social distancing prompted significant alterations in nursing practices [26]. Participants’ reports of task prioritization—such as focus on life-threatening issues—are consistent with findings by Albsoul et al. and Bruyneel et al., who identified an increase in patient volume and resource shortages lead to missed nursing care during COVID-19 crisis [27,28]. Consequently, resuscitative measures received the highest priority among multipronged care indicators, whereas supportive care, close patient monitoring, and management of adverse events ranked lowest [5,29–32]. This divergence may be attributable to the realities imposed by COVID-19, where resource scarcity and workplace pressures—along with emotional, educational, mobility, and hygienic needs of patients—necessitated the rationing of nursing care, thereby challenging the comprehensiveness of patient management [33]. The transition to task-focused care—emphasizing vital signs and medications over comprehensive patient needs—reflects the resource shortages that drive nurses to concentrate on immediate survival tasks during crises. The study’s focus on specific cancer-related priorities, such as chemotherapy management, introduces a level of specificity, demonstrating how patient reliance on critical interventions exacerbates this shift and highlighting the importance of tailored approaches in challenging circumstances. Patterson et al. similarly observed that task prioritization becomes pronounced in limited resources settings, supporting the current findings [34].

Nevertheless, interruptions or prioritization of care are not invariably negative; certain delays may, in fact, enhance patient safety, comfort, and the accuracy of nursing tasks [35]. Task prioritization can thus serve as an effective strategy for managing constrained resources [34]. When nurses are compelled to ration care, they tend to prioritize clinical tasks that directly affect the immediate well-being of the patient [36]. It is noteworthy that the omission of specific aspects of care among oncology nurses is a common phenomenon. These omissions often involve areas not directly related to therapeutic tasks but may carry increased risks and demand more meticulous planning due to their potential adverse outcomes [37].

Interrupted and delayed care due to emergencies and infection control aligns with Fernandez et al., who documented treatment postponements during COVID-19 due to shifting priorities and safety protocols [38]. The oncology context introduces unique risks, as chemotherapy interruptions, as noted by Participant No. 11, could worsen patient outcomes—a concern less emphasized in general nursing studies but critical here due to patients’ immunocompromised status.

This study expands existing literature by highlighting the particular context of oncology nursing, where the immunocompromised status of patients accentuates the consequences of missed care. Sessa et al. emphasized the vulnerability of cancer patients during pandemics and reported higher mortality rates associated with delays in care [39]. The current study’s finding—that deferred treatments have directly affected patient outcomes—supports this view and demonstrates that the harm caused by missed care within oncology settings entails unique implications. Contrasting with the previous cross-sectional studies which focus on the general nursing population, the phenomenological approach here vividly captures the lived experiences of oncology nurses. The absence of prior experience with emergent infectious diseases has influenced both the risk assessments and perceived competence levels of nurses. The unpreparedness for COVID-19 exposed a tendency among nurses to adhere to “doing things the way we always have,” which could have serious implications for pandemic preparedness—potentially neglecting the scientific evidence and best practices related to infectious disease management [40].

Our interpretation indicates that missed care behaviors among oncology nurses during the COVID-19 pandemic should not be understood simply as failures of performance but as deeply human responses to moral and existential conflicts. Nurses’ decisions to omit, delay, or shorten care reflect their attempts to reconcile ethical responsibilities with the constraints of an overburdened system. These behaviors are situated within a lived tension between the desire to provide holistic care and the necessity of self-protection and survival. From a Heideggerian perspective, such experiences represent a struggle of “being-in-care” — where the nurse’s professional identity is challenged by the impossibility of fulfilling the moral duty to care under crisis conditions.

### 5.2. Isolation in duty

The sub-theme of isolation in duty, encompassing insufficient backing, imbalance in power, stigmatized isolation, and unmet nurse needs, provides a novel contribution to the MNC literature.

The lack of supervisor support reported by participants echoes Shanafelt et al., who highlighted diminished leadership presence during the pandemic as a morale detractor [41]. This study’s finding that unsupported nurses reduced effort aligns with McClelland et al., linking inadequate backing to lower care quality. If nurses do not receive adequate support to uphold their physical, emotional, and psychological well-being as well as their professional integrity, their capacity to provide effective care and support to patients, families, and communities will be significantly impaired [42]. However, the oncology focus reveals a specific vulnerability, as supervisors’ reluctance to engage with COVID-positive units may reflect heightened risk perceptions not as prominent in other specialties.

Participating nurses acknowledged that teamwork among them faced challenges during the COVID-19 pandemic. according to studies, weaknesses in collaboration among team members were cited as a reason for incomplete care and MNC [43,44]. Indeed, the main challenges in fully executing the nursing role during this period included a lack of support from team members and tensions or communication disruptions within the nursing team [17,45,46]. Conversely, one study reported team cohesion during the COVID-19 pandemic [47]. This contradiction may be influenced by varying organizational cultures, particularly the team working atmosphere and standards of a healthy work environment. Process and structural interventions, such as improving teamwork and increasing the number of nurses, could significantly impact the reduction of MNC and service rationing [48]. The lack of opportunities for oncology nurses to participate in decision-making and management’s disregard for staff concerns are key factors contributing to MNC [49]. One study identified nurse managers’ abilities, leadership, and support for nurses as two influential factors in MNC [50]. During the pandemic, nurses perceived operational issues arising from power imbalances between management and frontline staff [51], which could serve as a contributing factor to MNC [9]. Nurse bullying, along with practice models that undermine the autonomy of clinical nurses, and a hierarchical, inflexible approach to nursing leadership, represent critical weaknesses in the nursing field [52]. Shared decision-making between frontline oncology nurses and leadership personnel is an essential component in oncology nursing, which is realized through addressing systemic issues such as staffing, educational opportunities, nurse well-being, and workplace bullying [53].

Also, Tensions with other clinical professionals in the oncology department negatively affect teamwork [54]. Studies show that the nurse-physician relationship and nurse involvement in hospital affairs are significantly associated with MNC [55]. Also, according to the Kalisch model, communication and teamwork are significant external factors that can lead to MNC [56]. In Iran, due to the traditional and unscientific structure of the healthcare system, the relationship between physicians and nurses is hierarchical, and nurses’ opinions regarding nursing care are often overlooked [57]. Power imbalances between nurses and physicians and hierarchical dynamics may hinder collaborative care during crises. Participant P10’s experience of forced care decisions by surgeons’ underscores this, suggesting oncology nurses face unique pressures due to patient complexity, a nuance less explored in broader literature.

In addition to the fading connections with patients and the treatment team, oncology nurses faced stigma as potential virus carriers, leading to feelings of rejection and social isolation Other studies have also shown that nurses experienced stigma both covertly and overtly during the pandemic [40,58,59]. The study’s identification of social stigma as a contributor to isolation (P7) is particularly significant, as it extends beyond the workplace to community interactions. Social stigma leading to isolation mirrors Ramaci et al., who documented healthcare workers’ alienation due to contagion fears [60]. This study’s oncology nurses faced amplified stigma due to their dual role with immunocompromised and COVID-positive patients, a distinct burden compared to findings in less specialized settings. Enhancing media education programs during the pandemic can reduce stigma and improve public awareness about nurses, ultimately boosting healthcare quality.

The current study’s emphasis on nurses’ unmet emotional, protectional, educational, and financial needs during the pandemic, which significantly impacted MNC. Factors related to the emotional health and overall well-being of nurses were consistently identified as the primary influences on incomplete nursing care during the pandemic [61]. The results of the studies indicated that the prevalence of post-traumatic stress disorder, anxiety, depression, and emotional exhaustion among nurses was high during the pandemic [62]. Given the increase in negative emotions throughout the pandemic, providing mental health support should be an essential component of healthcare services for providers during this crisis to ensure effective nursing care and management of the pandemic [63,64]. Previous studies have also reported that perceived adequacy of PPE is a strong predictor of MNC, with nurses who perceived sufficient PPE in their units reporting fewer instances of missed care [65]. Thus, the perceived lack of PPE emerged as a contributing factor to the concerns and fears experienced by nurses [66]. When the physical and emotional safety of nurses is jeopardized, ethical dilemmas emerge. As professionals, nurses hold responsibilities not only towards their patients but also towards themselves, their families, colleagues, and the broader society. Should the working environment undermine this protective framework, moral distress may arise, negatively impacting communication and, consequently, the nurse-patient relationship [67]. Findings from studies also provide evidence for the need to develop the roles of clinical nurse specialists and professional development training programs in oncology due to their potential benefits for nurses, physicians, patients, family members, and the healthcare system [68]. Consequently, enhancing the education and specialization of nurses within healthcare teams, with a particular focus on strengthening certain personality traits to transform them into more effective and efficient therapeutic agents, is of utmost importance [69]. Government acknowledgment of specialized oncology education and involvement in national cancer control planning play a crucial role in strengthening and maintaining the essential workforce of oncology nursing [52]. This matter holds particular significance during pandemics. Enhancing the roles of oncology nurses, coupled with the requisite training and competencies to formally recognize them as care managers across the continuum of cancer care, has the potential to improve the quality of cancer treatment [70]. Improving safety conditions and providing appropriate equipment can help reduce anxiety, enhance the sense of value among nurses, and ultimately improve the quality of care delivered.

Ultimately, income satisfaction and organizational support were among the factors associated with levels of MNC during the COVID-19 pandemic [17]. Disparities in pay and rewards for nurses on the front lines of the COVID-19 battle created a non-supportive work environment that negatively impacted the quality of nursing care [4]. Nurses participating in a study expressed feelings of unfairness due to inequalities in work and benefits compared to other healthcare providers, including physicians, highlighting the disregard for their professional roles in combating the virus [71]. Fernández et al. also stated that nurses require support from governments, policymakers, and nursing organizations to implement measures in their favor during and after a pandemic [38]. Success in new work patterns necessitated supportive relationships, care, and understanding from colleagues and upper management to alleviate negative feelings [72,73].

In general, it is said that results of a study showed that psychological interventions increase participation and reduce conflict factors within the treatment team. Group therapy focused on changing individuals’ responses to stress-inducing situations can engender more adaptive strategies, enabling caregivers, oncology specialists, and nurses to better assist patients and thereby improve their quality of life [74]. Therefore, it is evident that meeting the social and administrative support requirements of oncology nurses within healthcare teams and at the institutional level is crucial.

We interpret the sub-theme of “Isolation in Duty” as a profound manifestation of moral and relational disconnection. The nurses’ experiences reveal that isolation was not only physical but existential, eroding their sense of belonging and professional authenticity. The imbalance of power and lack of managerial and social support reflect an organizational environment that diminished the meaning of care. This isolation disrupted the relational essence of nursing and created an inner sense of alienation, which can be interpreted as a breakdown of the shared “world of care.” From the authors’ perspective, this isolation points to the urgent need for moral resilience, ethical leadership, and institutional empathy within oncology settings, especially during large-scale crises.

### 5.3. Disruption of care canvas

The disruption of care canvas, driven by a storm of responsibilities, intensified environmental issues, and forced adaptation, underscores systemic barriers to effective nursing care. Oncology nurses faced challenges arising from the transformation in the care environment. Participants’ reports of Increase in the number of patients, emergency within the unit or deterioration of one of the assigned patients, various interruptions during work and high workload align with Kim et al., and Obregón et al., who noted the challenges of caring for patients during pandemics [26,75]. Additionally, the unpredictable situations reported by our participants have also been documented in studies by Safdari et al, and Hosseini et al, [4,5]. Unexpected rise in patient volume and/or acuity in the unit and urgent patient situations were also issues related to missed nursing care that have been reported in other studies during the pandemic [8,23,31,76–80]. This study’s finding of untrained staff managing chemotherapy highlights a unique oncology challenge, extending beyond general shortages to skill mismatches, an area underexplored in prior research.

The shortages of material and human resources reported by oncology nurses in this study align with other studies conducted in Iran during the pandemic, identified as factors contributing to the missed nursing care [4,5,12,81] and reflecting a broader, underlying issue across the healthcare system. In another study, the results indicated that during the COVID-19 pandemic, Iran’s healthcare system also revealed organizational, resource-related, and managerial challenges and barrier [82]. Blackman et al. also identified workload intensity and workload predictability as key factors associated with missed nursing care prior to the pandemic [83]. The ability of healthcare facilities to act and respond underwent significant changes during the global pandemic triggered by the COVID-19 virus [84]. According to studies, the emergence of new priorities within the healthcare system and the increased demand for care were identified as reasons for unfinished nursing care during the pandemic [26,85]. Different types of PPE, and at times the shortage of PPE, may exert additional pressure on nursing staff, leading to increased anxiety regarding both their potential infection and the risk of transmitting the virus to other patients or family members, particularly those on the front lines [86]. The oncology setting highlights a higher-risk environment and underscores an urgent need for both material and human resources, especially in crisis conditions.

Also, participants discussed the obligation to adapt to issues arising from changes in the work environment during the COVID-19 pandemic. Excessive workload, the requirement to work in isolation, the inability to move items in and out of isolation rooms without donning and doffing personal protective equipment (PPE), stress from infection risks [25,26,87], and were identified by nurses as causes of MNC during the COVID-19 pandemic. The emotional impact of disrupted care environments, such as P8’s difficulty assessing patients through masks, introduces a human element not fully explored in prior research. While Kalisch et al. [56] discuss the economic and ethical implications of MNC, they do not address the emotional toll of environmental constraints. The current study’s findings thus bridge a gap in the literature by linking systemic failures to nurses’ lived experiences, supporting calls by Phillips et al. for research to improve cancer care during pandemics [22].

From an interpretive standpoint, the “Disruption of Care Canvas” symbolizes the collapse of the nurses’ familiar world of care, where environmental, temporal, and relational dimensions of nursing practice were fractured. The pandemic transformed the caring space from one of mutual connection to one of constant vigilance and fragmentation. The nurses’ forced adaptation to changing protocols and restricted physical proximity can be seen as a transformation of their caring embodiment — the body as the medium of empathy and presence became shielded and distant. This interpretation highlights how systemic breakdowns are experienced not only operationally but existentially, affecting nurses’ sense of purpose and professional identity.

The study’s findings contribute to nursing theories related to care quality and nurse well-being, particularly Watson’s Theory of Human Caring and the Missed Nursing Care Model. Theoretically, these findings can be applied to refine nursing frameworks by integrating pandemic-specific challenges. For example, Watson’s theory could be adapted to include strategies for maintaining caring relationships under resource constraints, while the Missed Nursing Care Model could incorporate social stigma as a nurse-related factor. These adaptations would enhance the applicability of these theories to oncology nursing during outbreaks, providing a foundation for future research and practice.

Overall, our interpretive analysis suggests that missed nursing care during the pandemic reflects more than a deficit in healthcare systems — it represents an existential struggle to preserve the essence of caring within a fragmented professional world. The fading connections described by oncology nurses embody the loss of relational, moral, and structural harmony that defines authentic nursing practice. By interpreting these experiences through a phenomenological lens, the authors emphasize the need to view nursing not only as a technical act but as an existential commitment to human connection, which must be supported through empathy-based leadership, organizational solidarity, and cultural recognition of nurses’ moral labor.

## 6. Limitation

This phenomenological study provides a novel contribution by exploring the lived experiences of oncology nurses regarding missed nursing care during the COVID-19 pandemic, a topic not previously reported. Its main strength lies in offering in-depth insights into how nurses experienced, perceived, and navigated missed care in oncology wards under crisis conditions. The hermeneutic phenomenological approach allowed for a deeper understanding of the meaning of missed nursing care, capturing both the emotional and ethical dimensions of practice. Participants’ firsthand experiences, as frontline oncology nurses during the pandemic, enriched the study’s findings and enhanced their credibility.

However, several limitations should be considered. First, the study was conducted in two cities in Iran, which may limit generalizability, although similar experiences are likely in comparable settings. Second, only nurses’ perspectives were included, excluding patients and supervisors, which may overlook broader systemic or interprofessional dynamics. Third, potential biases such as social desirability and recall bias could have influenced participants’ reporting of missed care, although conducting two interviews per participant helped mitigate recall bias. Additionally, the study did not quantify the frequency or impact of specific missed care behaviors, limiting its ability to prioritize interventions.

The interpretive nature of hermeneutic phenomenology also introduces subjectivity, as researchers’ perspectives inevitably shape the findings. Measures such as member checking were employed to enhance trustworthiness. Despite these limitations, the study provides valuable insights into the relational, emotional, and hidden boundaries of oncology nursing during a health crisis, offering evidence that can inform future research, policy, and clinical strategies to better support healthcare professionals in similar circumstances.

Future research should employ both qualitative and longitudinal designs across diverse cultural and clinical settings to examine nurses’ experiences, interprofessional dynamics, patient perspectives, and the long-term impacts of missed nursing care, with a focus on preparedness, risk perception, and adherence to care practices in specialized areas such as oncology.

## 7. Conclusion

This study examines oncology nurses’ experiences regarding MNC during the COVID-19 pandemic. When human and professional relationships are compromised, the essence of nursing care is lost, and it risks being reduced to a mechanical, task-focused practice. The findings highlight that poor communication encapsulates disrupted relationships with patients, care teams and environmental deficiencies including inadequate infrastructure and staffing shortages significantly impacted the quality of care provided. Furthermore, the study underscores the importance of teamwork and effective leadership in mitigating these challenges, as well as the need for systemic changes to address the hierarchical structures within healthcare that often marginalize nursing contributions.

The stigma associated with being a healthcare worker during the pandemic further exacerbated feelings of isolation among nurses, underscoring the need for greater public awareness and support. Additionally, addressing the unmet emotional, educational, and financial needs of oncology nurses is crucial for enhancing their well-being and ensuring the delivery of comprehensive care. professional frustration may lead some nurses to leave the profession, exacerbating the nursing shortage during pandemics. These findings underscore the systemic and emotional barriers oncology nurses navigated, highlighting the urgent need for supportive interventions to mitigate MNC and enhance patient outcomes in crisis settings. These risks must be carefully considered, and the nursing profession should not ignore them.

Many strategies to enhance the quality of nursing services and reduce missed care require relatively simple and practical actions. Comprehensive support for oncology nurses during the pandemic and ensuring their sense of security while providing care should be fundamental principles in any sensitive clinical environment. Future planning should include the establishment of consistent processes for emerging infectious diseases. This necessitates innovation and changes in existing approaches to more effectively address new challenges. We must listen to the shared experiences in this phenomenological study and respond by providing the necessary supportive resources to improve nursing performance and care.

## 8. Nursing policy implications

Nursing managers must enhance support for oncology nurses during pandemics by recognizing their equal value to other healthcare professionals and granting them greater autonomy in patient care decisions. This includes developing specialized nursing knowledge and ensuring the allocation of adequate, high-quality safety equipment, as many nurses have reported deficiencies. Workloads must align with appropriate staffing ratios to allow sufficient time for comprehensive care. Establishing suitable care environments before crises and creating policies to reduce burnout and provide financial support is essential. Increasing the number of practicing oncology nurses during health crises and prioritizing ongoing education in oncology nursing is vital. Leadership training for supervisors could foster empathetic communication, countering the inadequate backing reported. Finally, addressing power imbalances with physicians requires interprofessional collaboration initiatives, such as team-building workshops, to enhance care coordination. By adopting these strategies, nursing managers can significantly improve the effectiveness of oncology nursing teams during health crises.
